# Latent Classes of Personality Traits and Their Relationship with Workplace Bullying among Acute and Critical Care Nurses

**DOI:** 10.1155/2024/3238636

**Published:** 2024-03-15

**Authors:** Meng Sun, Jing Han, Ying Qiao, Juan Wang, Mei Jiang, Min Zhang

**Affiliations:** ^1^Department of Emergency Intensive Care Unit, Qilu Hospital of Shandong University, Jinan, Shandong Province, China; ^2^Department of Endocrinology and Metabolism, Qilu Hospital of Shandong University, Jinan, Shandong Province, China; ^3^Mental Health Center, Hangzhou Seventh People's Hospital, School of Brain Science and Brain Medicine, Zhejiang University School of Medicine, Hangzhou, Zhejiang, China; ^4^Department of Emergency, Qilu Hospital of Shandong University, Jinan, Shandong Province, China; ^5^Department of Nursing Department, Qilu Hospital of Shandong University, Jinan, Shandong Province, China

## Abstract

**Objective:**

To identify the latent classes of personality traits among nurses in acute and critical care departments, as well as the relationship between latent classes of different personality traits and workplace bullying.

**Methods:**

A total of 245 nurses working in the acute and critical care department at a 3 A-grade hospital in Shandong Province, China, were recruited by convenient sampling. The Chinese Big Five Personality Inventory brief version was used to assess personality traits, and the Negative Acts Questionnaire was used to measure workplace bullying. Latent profile analysis was used to identify the latent classes of personality traits. Multiple linear regression analysis was used to examine the relationship between latent classes of personality traits and workplace bullying.

**Results:**

Four latent classes of personality traits among acute and critical care nurses were identified, namely, the negative group (49.0%), flexible group (16.0%), neurotic group (18.1%), and stable group (16.9%), respectively. Compared with the neurotic group, the negative group (*B* = −6.227, *P* < 0.05), stable group (*B* = −16.562, *P* < 0.001), and flexible group (*B* = −19.208, *P* < 0.001) experienced less workplace bullying.

**Conclusion:**

Our findings explore latent classes of personality traits among acute and critical care nurses, identify subgroups susceptible to workplace bullying, and suggest the development of appropriate interventions to reduce workplace bullying. *Implications for Nursing Management*. Hospital managers can identify nurses who are prone to workplace bullying based on their personality traits and provide them with psychological counseling services and psychological healing groups to help them establish good interpersonal relationships and maintain their mental and physical health.

## 1. Introduction

Workplace bullying, as a workplace stressor, refers to the negative behaviors of work-related harassment, offense, and exclusion that an individual frequently and repeatedly experiences in the workplace for more than six months (at least once a week) [1]. Research has shown that workplace bullying is more common and severe for nurses than other professions [2], especially for acute and critical care nurses who work in the emergency room and intensive care unit [3]. Workplace bullying not only has adverse effects on the physical and mental health of nurses [4], but also causes job burnout [5], increases the turnover tendency of nurses [6], and even affects the quality of nursing care and threatens the life safety of patients [6]. Therefore, more attention should be paid to identifying risk factors for workplace bullying among acute and critical care nurses.

Personality traits refer to the stable and unique psychological and behavioral patterns that distinguish oneself from others and are shaped under the combined action of congenital heredity and acquired environmental conditions. Previous studies have shown that personality traits, as a typical variable reflecting individual differences, play a significant role in predicting individuals' exposure to workplace bullying [7–9]. For example, Balducci et al. [10] found that neuroticism was positively correlated with workplace bullying among 609 public sector employees in Italy. However, most studies focus on the relationship between a certain personality trait (e.g., neuroticism and agreeableness) and workplace bullying from a variable-centered perspective and neglect that different types of personality traits can exist simultaneously.

Latent profile analysis (LPA) [11] is a person-centered statistical analysis identifying different subgroups within populations that share certain outward characteristics. LPA can be used to analyze the relationship between personality traits and workplace bullying when taking different types of personality traits into consideration simultaneously. Studies have shown that individuals with the same level of personality traits show different characteristics in different dimensions [12, 13]. However, the relationship between the latent classes of personality traits and workplace bullying remains underinvestigated.

Therefore, this study aimed to apply LPA to explore different potential classes of personality traits of acute and critical care nurses and to analyze their relationship with workplace bullying.

## 2. Materials and Methods

### 2.1. Study Participants

From April to September 2022, a total of 245 acute and critical care nurses in a 3 A-grade hospital in Shandong Province were selected as participants by the convenience sampling method. The inclusion criteria for acute and critical care nurses were as follows: (1) registered nurse with valid license; (2) working in the acute and critical care departments for more than 3 months (three months is a probation period); and (3) informed consent and voluntary participation. The exclusion criteria were: not having been employed as a nurse in this hospital.

### 2.2. Procedures

In this study, the online questionnaire collection was carried out through the “Wenjuanxing” platform with the support of the consent of the nursing department and head nurses of all departments in the hospital. The study followed the principles of voluntary participation and informed consent with the ethics approval number 2020-R-061. Before completing the questionnaire, the objective and procedure of the questionnaire were detailed. Finally, 245 valid questionnaires were collected.

### 2.3. Study Tools

#### 2.3.1. Sociodemographic Characteristics

Personal characteristics include sex, age, level of education, and marital status; job-related characteristics include average monthly income, employment relationship, professional title, working years, night shift involvement, number of monthly night shifts, and weekly working hours.

#### 2.3.2. Chinese Big Five Personality Inventory Brief Version (CBF-PI-B)

The CBF-PI-B [14] was used to assess personality traits by acute and critical care nurses. It comprises 40 items and can be classified into 5 dimensions (i.e., neuroticism, extroversion, openness, agreeableness, and conscientiousness). Each item was rated on a 5-point scale, with a point from 1 to 5 indicating “strongly disagree” to “strongly agree.” The higher the total score of each dimension is, the more obvious the corresponding personality characteristics are. Cronbach's alpha of the total scale in this study was 0.878.

#### 2.3.3. Negative Acts Questionnaire (NAQ)

The NAQ was developed by Einarsen and Notelaers [15] and was translated into Chinese by Hongjing Xun [16] in 2012. It has been widely used in the measurement of workplace bullying suffered by Chinese nurses. The NAQ has 22 items and includes 3 dimensions (i.e., individual-related negative acts, work-related negative acts, and organizational injustice). A 5-point scale was used to rate each item, with a point from 1 to 5 indicating “never,” “occasionally,” “monthly,” “weekly,” and “daily,” respectively. The higher the total score is, the higher the level of workplace bullying nurses experience. Cronbach's alpha of the total scale in this study was 0.980.

### 2.4. Statistical Methods

Firstly, for continuous variables conforming to a normal distribution, mean and standard deviation (SD) were used for description; for non-normal distribution data, median and interquartile range (IQR) were used for description; for categorical variables, frequency and percentage were used for description.

Secondly, LPA was conducted to identify latent profiles of personality traits. We started with a one-profile model and gradually increased the number of profiles until the optimal model was identified. The model was comprehensively evaluated according to Entropy, Bootstrap likelihood ratio test (BLRT), Lo–Mendell–Rubin adjusted likelihood ratio test (LMR), Akaike information criteria (AIC), Bayesian information criteria (BIC), sample-size-adjusted Bayesian information criterion (aBIC). The smaller the AIC, BIC, and aBIC values are, the better the fitting degree is. Entropy ranges from 0 to 1, with higher values (value ≥ 0.8) indicating a clearer classification [17]. When the results of LMR and BLRT are <0.05, it indicates that the K class model is superior to K-1 class model [18].

Thirdly, the chi-square test was used to analyze sociodemographic differences of potential personality traits among nurses in acute and critical care units.

Fourthly, the Kruskal–Wallis test was used to explore the relationship between the latent classes of personality traits of nurses and workplace bullying.

Fifthly, the Wilcoxon–Mann–Whitney test and Kruskal–Wallis test were used to analyze the differences in workplace bullying among acute and critical care nurses with different sociodemographic characteristics.

Finally, multiple linear regression was conducted to analyze the relationship between latent profiles of personality traits and workplace bullying, and statistically significant demographic variables in the univariate analysis were controlled as covariates. The significance level was set at *α* = 0.05 (two sides). SPSS 25.0 and Mplus 8.0 were used for statistical analyses.

## 3. Results

### 3.1. Latent Profiles of Personality Traits among Acute and Critical Care Nurses

As shown in Table 1, six models were obtained by fitting from one to six latent classes, respectively. The entropy values of the 2-class to 6-class models were >0.8, indicating good accuracy of these models. As the number of latent classes increased, AIC, BIC, and aBIC gradually decreased. In the model of Class 4, the *P* values of both LMR and BLRT were significant; indicating that the model of Class 4 was superior to the model of Class 3, but the *P* value of LMR of the model of Class 5 was nonsignificant. Therefore, the model of Class 4 was determined as the optimal model.

To verify the reliability of the analysis results of the above latent classes, the average posterior probability of different models is shown in Table 2. The results showed that the average posterior probability (column) of the four latent classes (row) of nurses' personality traits ranged from 0.786 to 0.954, indicating that the model fitting results of the four latent classes were reliable.

**Figure 1:**
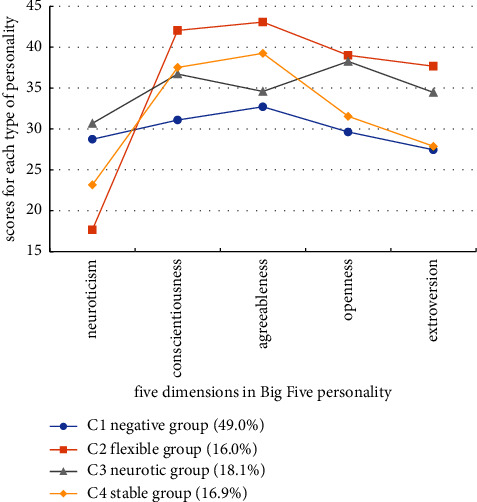
Latent classes of personality traits in acute and critical care nurses.

The first class (49.0%) called the “C1 negative group” scored higher on the neurotic dimension than the C2 flexible group and the C4 stable group but lower than the C3 neurotic group and the lowest on the dimensions of conscientiousness, agreeableness, openness, and extroversion. The second class (16.0%) had the lowest score on the dimension of neuroticism and the highest score on other dimensions and was named as “C2 flexible group.” The third class (18.1%) had the highest score on the dimension of neuroticism, moderate scores on the dimensions of conscientiousness, agreeableness, openness, and extroversion, and was named as “C3 neurotic group.” The final class (16.9%) had low scores in neuroticism, openness, and extroversion and high scores in conscientiousness and agreeableness, indicating conservative personality and stable mood, and was labeled as “C4 stable group” (Figure 1).

### 3.2. The Differences in Sociodemographic Characteristics of Nurses in Different Latent Classes of Personality Traits

Univariate analysis results showed that there were statistically significant differences (*P* < 0.05) in age, professional title, and monthly night shift number of nurses in different latent classes of personality traits (Table 3). Flexible group were older than neurotic group nurses. Compared with acute and critical care nurses of high professional titles, those with low professional titles accounted for a higher proportion in negative group and neurotic group. Compared with those without night shifts, acute and critical care nurses with night shifts accounted for a higher proportion in negative group and neurotic group.

### 3.3. Relationship between Latent Classes of Personality Traits and Workplace Bullying among Acute and Critical Care Nurses

#### 3.3.1. Disparity between Latent Classes of Personality Traits and Workplace Bullying among Acute and Critical Care Nurses

As shown in Table 4, the dimensions and total scores of workplace bullying showed significant differences in the latent classes of personality traits among acute and critical care nurses (*P* < 0.001). Post hoc analysis showed that the C1 negative group and the C3 neurotic group scored higher than the flexible group and stable group in the two dimensions of individual-related negative acts and work-related negative acts and the total score of negative acts. In the dimension of organizational injustice, the neurotic group scored higher than the flexible group and the stable group, and the negative group scored higher than the flexible group.

#### 3.3.2. Wilcoxon–Mann–Whitney Test and Kruskal–Wallis Test Were Used to Analyze the Differences in Workplace Bullying among Acute and Critical Care Nurses with Different Sociodemographic Characteristics

The normality test of workplace bullying suffered by nurses showed a skewed distribution. Therefore, the Wilcoxon–Mann–Whitney test and Kruskal–Wallis test were used to analyze the relationship between sociodemographic characteristics and workplace bullying. The results showed that age, marital status, weekly working hours, and average monthly income of participating nurses correlated with workplace bullying significantly (*P* < 0.05).

#### 3.3.3. Multiple Linear Regression Was Conducted to Analyze the Relationship between Latent Profiles of Personality Traits and Workplace Bullying

Multiple linear regression analysis showed that after controlling for age, marital status, weekly working hours, and average monthly income, compared with the neurotic group, the negative group (*B* = −6.227, *P* < 0.05), stable group (*B* = −16.562, *P* < 0.001), and flexible type (*B* = −19.208, *P* < 0.001) reported lower levels of workplace bullying (Table 5).

## 4. Discussion

This study found four heterogeneous groups of personality traits among acute and critical care nurses, including negative, neurotic, flexible, and stable groups. The stable group (16.9%) was introverted but rigorous, got along well with others, and had stable emotions. The flexible group (16.0%) was rigorous, extroverted, highly tolerant and empathetic to others, and able to handle interpersonal relations flexibly. Although the stable and flexible groups had been identified in previous studies [12, 13], this study still proposed two new groups of personality traits, namely, negative group and neurotic group. The negative group (49.0%) was introverted, indifferent to others with low social participation, and may show a negative attitude at work. The neurotic group (18.1%) was overly sensitive, lowly tolerant to others, prone to anxiety, and had an unstable personality. The reason may be related to the job content of emergency and critical care departments. Acute and critical care nurses face and rescue more critically ill patients with more workload, working time, work pressure, and night shifts, which may induce negative attitudes and negative emotions [19, 20], so there are the negative group and neurotic group.

In this study, there were significant differences in age, professional title, and number of monthly night shifts among latent classes of personality traits. Flexible group nurses were older than neurotic group nurses. Compared with acute and critical care nurses of high professional titles, those with low professional titles accounted for a higher proportion in the negative group and neurotic group. Compared with those without night shifts, acute and critical care nurses with night shifts accounted for a higher proportion in the negative group and neurotic group, which was consistent with the results of previous studies [12]. It is worth noting that the negative group and neurotic group were prone to anxiety, depression, and other negative emotions [21] and more likely to have career burnout [12]. Consistently, this study revealed that the negative group and the neurotic group exhibited higher overall scores of workplace bullying when compared to both the flexible group and the stable group, indicating a greater susceptibility to workplace bullying. Therefore, nursing managers should pay more attention to negative and neurotic nurses.

Multiple linear regression results showed that compared with the neurotic group, the negative, stable, and flexible groups all suffered from less workplace bullying considering nurses' sociodemographic characteristics and working hours as the potential confounders. This was consistent with the results of previous studies that personality traits are closely related to workplace bullying [9, 22]. This study found that nurses prone to workplace bullying did not have a single personality trait but a personality type composed of several personality traits. Individuals with low neuroticism, high conscientiousness, high agreeableness, high extroversion, and high openness and individuals with low neuroticism, high conscientiousness, high agreeableness, low extroversion, and low openness were not prone to workplace bullying [23]. According to the Victim Precipitation Theory [24], certain characteristics or behaviors exhibited by people may become their weaknesses, attract others' encroachment, and make them the targets to be hurt. Acute and critical care nurses in the neurotic group are emotionally unstable, difficult to get along with others and easy to be isolated by colleagues, and become the target of bullying. Nurses in both the flexible group and stable group are introverted or extroverted and can maintain a responsible and rigorous attitude with less negative emotions and better deal with interpersonal relationships; hence, they are less likely to be bullied by colleagues [25].

## 5. Limitations

There are several limitations of the present study. Firstly, using a cross-sectional design, this study could not predict the longitudinal trajectory of nurses' exposure to workplace bullying, nor could it draw a causal relationship between potential categories of personality and workplace bullying, which needs to be confirmed by more empirical studies in the future. Secondly, the representativeness of the existing study participants is limited. This study only surveyed nurses in the emergency critical care department of a 3 A-grade hospital, and the sample size was small. Third, confounding factors such as working environment, organizational problems and other factors may not be fully taken into account in this study; it is suggested that more comprehensive confounding factors should be included in future studies for analysis.

## 6. Conclusion

There are four subtypes of personality traits among acute and critical care nurses: the negative group, flexible group, neurotic group, and stable group. Nurses in the neurotic group are more likely to suffer from workplace bullying. It is necessary to identify the personality traits of nurses and develop interventions to reduce workplace bullying.

## 7. Implications for Nursing Management

There are several implications for nursing management. First, hospital managers should pay attention to neurotic and negative nurses who are prone to workplace bullying. Although their personality characteristics are stable and not easy to change, managers should take active and effective measures to help them, such as setting up psychological counseling departments and psychological healing teams in the department to help nurses who have been bullied get rid of the adverse effects. Guide them to build good interpersonal relationships and maintain their mental and physical health. Second, hospital managers can help nurses to understand and cope with workplace bullying by organizing training related to workplace bullying, and carry out targeted training for nurses with different personality characteristics and different ages, so as to improve the awareness and handling ability of nurses. Third, department leaders can form a positive nursing work atmosphere by organizing team building activities or constructing department culture. At the same time, they can set a team work mode, comprehensively consider the characteristics of nurses, work ability, and other factors to assign team members, form a mutual help and harmonious group work atmosphere, and increase the ability to resist workplace bullying. In addition, hospital managers can take the potential characteristics of nurses' personality traits as a reference index for the selection of nursing talents and the allocation of departments, assign nurses with different personalities to suitable departments, and select nursing talents more suitable for emergency and critical department posts [26].

## Figures and Tables

**Table 1:** Latent profile analysis of personality traits among acute and critical care nurses.

Model	Loglikelihood	AIC	BIC	aBIC	Entropy	LMR	BLRT
1	−3858.521	7737.043	7772.055	7740.356	…	…	…
2	−3693.684	7419.369	7475.389	7424.670	0.847	0.0005	0.0000
3	−3652.435	7348.870	7425.897	7356.159	0.814	0.2567	0.0000
4	−3619.410	7294.819	7392.854	7304.097	0.815	0.0458	0.0000
5	−3590.718	7249.436	7368.479	7260.701	0.815	0.3042	0.0000
6	−3570.915	7221.830	7361.880	7235.083	0.837	0.2648	0.0000

*Note*. AIC: Akaike information criteria, BIC: Bayesian information criteria, aBIC: sample-size-adjusted Bayesian information criterion, LMR: Lo–Mendell–Rubin adjusted likelihood ratio test, BLRT: bootstrap likelihood ratio test.

**Table 2:** Average posterior probability of latent classes of personality traits in acute and critical care nurses.

Latent Class	1	2	3	4
1	0.954	0	0.02	0.027
2	0	0.943	0.042	0.015
3	0.092	0.017	0.855	0.036
4	0.133	0.03	0.051	0.786

**Table 3:** Distribution comparison of latent classes of personality traits among acute and critical care nurses with different demographic characteristics (*n* (%)).

Variables		C1 negative group	C2 flexible group	C3 neurotic group	C4 Stable group	*F/ χ* ^2^ value	*P* value
*General demographic characteristics*							
Age (year)	30.26 ± 4.776		31.90 ± 5.816	28.59 ± 4.495	30.45 ± 5.187	3.082	**0.028**
Sex						7.012	0.072
Male	27 (49.1%)		6 (10.9%)	16 (29.1%)	6 (10.9%)		
Female	97 (51.1%)		33 (17.4%)	28 (14.7%)	32 (16.8%)		
Marital status						5.494	0.139
Married	77 (50.3%)		30 (19.6%)	23 (15.0%)	23 (15.0%)		
Other	47 (51.1%)		9 (9.8%)	21 (22.8%)	15 (16.3%)		
Level of education						6.256	0.066
Bachelor degree or below	118 (50.6%)		34 (14.6%)	43 (18.5%)	38 (16.3%)		
Master degree or above	6 (50.0%)		5 (41.7%)	1 (8.3%)	0 (0.0%)		
Average monthly income (RMB)						13.629	0.118
<4000	5 (38.5%)		2 (15.4%)	2 (15.4%)	4 (30.8%)		
4000–8000	41 (50.6%)		12 (14.8%)	17 (21.0%)	11 (13.6%)		
8000–10000	50 (61.7%)		7 (8.6%)	13 (16.0%)	11 (13.6%)		
>10000	28 (40.0%)		18 (25.7%)	12 (17.1%)	12 (17.1%)		
*Work-related characteristics*							
Working years						6.142	0.407
≤5		45 (48.4%)	11 (11.8%)	23 (24.7%)	14 (15.1%)		
6–10		46 (50.5%)	16 (17.6%)	14 (15.4%)	15 (16.5%)		
≥11		33 (54.1%)	12 (19.7%)	7 (11.5%)	9 (14.8%)		
Employment form						5.415	0.132
Tenure and personnel agency		10 (35.7%)	8 (28.6%)	4 (14.3%)	6 (21.4%)		
Contract and labor dispatch		114 (52.5%)	31 (14.3%)	40 (18.4%)	32 (14.7%)		
Professional title						13.137	**0.041**
Nurse		27 (47.4%)	4 (7.0%)	17 (29.8%)	9 (15.8%)		
Senior nurse		70 (53.8%)	21 (16.2%)	21 (16.2%)	18 (13.8%)		
Chief nurse		27 (46.6%)	14 (24.1%)	6 (10.3%)	11 (19.0%)		
Weekly working hours						7.454	0.281
≤40		35 (42.7%)	15 (18.3%)	14 (17.1%)	18 (22.0%)		
41–45		66 (56.4%)	15 (12.8%)	20 (17.1%)	16 (13.7%)		
≥46		23 (50.0%)	9 (19.6%)	10 (21.7%)	4 (8.7%)		
Number of monthly night shifts						34.357	**<0.001**
None		10 (28.6%)	13 (37.1%)	3 (8.6%)	9 (25.7%)		
1–9		45 (57.7%)	17 (21.8%)	8 (10.3%)	8 (10.3%)		
≥10		69 (52.3%)	9 (6.8%)	33 (25.0%)	21 (15.9%)		

*Note*. *P*-values in bold are statistically significant for an alpha of 0.05.

**Table 4:** Disparity between latent classes of personality traits and workplace bullying among acute and critical care nurses.

Variables	C1	C2	C3	C4	*H* value	Comparison
Individual-related negative acts	15 (11, 20.5)	9 (9, 12)	15.5 (11, 28)	11 (9, 14.25)	38.256^*∗∗∗*^	C1 > C2, C4 C3 > C2, C4

Work-related negative acts	16 (12, 21)	10 (9, 12)	15.5 (12, 26)	11 (10, 14.25)	50.095^*∗∗∗*^	C1 > C2, C4 C3 > C2, C4

Organizational injustice	8 (7, 11)	6 (4, 8)	9 (6.25, 14)	7.5 (5, 9)	24.720^*∗∗∗*^	C1 > C2 C3 > C2, C4

Scores of negative acts	39 (31, 53.5)	24 (23, 33)	41.5 (31, 68.75)	32 (24, 36.25)	41.077^*∗∗∗*^	C1 > C2, C4 C3 > C2, C4

*Note*. ^*∗∗∗*^*P* < 0.001; C1: negative group; C2: flexible group; C3: neurotic group; C4: stable group.

**Table 5:** Relationship between latent classes of personality traits and workplace bullying among acute and critical care nurses.

Variables	*B*	SE	*t*	*P* value	95% CI
Classes of psychological violence					
Neurotic group	Reference group				
Negative group	−6.523	3.156	−2.067	**0.040**	(−12.741, −0.305)
Stable group	−16.051	4.015	−3.998	<**0.001**	(−23.961, −8.142)
Flexible group	−18.907	4.011	−4.713	**<0.001**	(−26.810, −11.004)
Age	0.167	0.324	0.516	0.607	(−0.472, −0.806)
Marital status					
Married	Reference group				
Others	−1.650	3.111	−0.530	0.569	(−7.779, 4.480)
Weekly working hours					
<40	Reference group				
41–45	3.456	2.660	1.299	0.195	(−1.784, 8.697)
≥46	3.463	3.431	1.009	0.314	(−3.296, 10.223)
Average monthly income (RMB)					
<4000	Reference group				
4000–8000	1.765	5.383	0.328	0.743	(−8.839, 12.370)
8000–10000	7.539	5.667	1.330	0.185	(−3.627, 18.705)
>10000	4.283	5.811	0.737	0.462	(−7.166, 15.732)

*Note*. *P*-values in bold are statistically significant for an alpha of 0.05.

## Data Availability

The data used to support the findings of this study are available from the corresponding author upon reasonable request.
